# Anthrax Toxin: A Pioneer of Targeted Protein Toxins

**DOI:** 10.3390/toxins17110533

**Published:** 2025-10-29

**Authors:** Sandy Richter, Gudula Schmidt

**Affiliations:** Institute for Experimental and Clinical Pharmacology and Toxicology, Faculty of Medicine, University of Freiburg, Albertstr. 25, 79104 Freiburg, Germany; dr.sandyrichter@gmail.com

**Keywords:** anthrax toxin, cell targeting, protein transport

## Abstract

With anthrax toxin as a pioneer, several bacterial toxins have been engineered to deliver proteins of interest into the cytosol of specific mammalian cells. Such targeted protein toxins combine the ability to deliver cargo molecules into cells with designed receptor interaction for targeting selected cells. This review summarizes the actual knowledge of modified anthrax toxin. Moreover, the significant efforts which have been made to utilize other bacterial toxins are discussed. The targeted protein toxins described in this review include single-chain toxins, pore-forming toxins, and injection machineries.

## 1. Introduction

Pathogenic bacteria produce and secrete diverse protein toxins. As virulence factors, these toxins play essential roles in disease progression. These toxins influence the immune system and open epithelial barriers, allowing the respective bacteria to reach deeper tissues, to name only a few effects among others. Most of the protein toxins have the ability to bind to mammalian cells and to enter the cytosol where they modulate or modify important signaling molecules. Therefore, these toxins are composed of several independently acting domains, designated as A for activity and B for binding and uptake. The domain order of the AB toxins is not always the same. Some toxins encompass an N-terminal binding and translocation domain with the catalytic domain present at the C-terminus, while others are oriented vice versa. A third class of toxins is composed of separated A and B domains which are assembled non-stoichiometrically. *Bacillus anthracis* anthrax toxin belongs to this class of toxins composed of two separated proteins.

## 2. Anthrax Toxins

**Figure 1:**
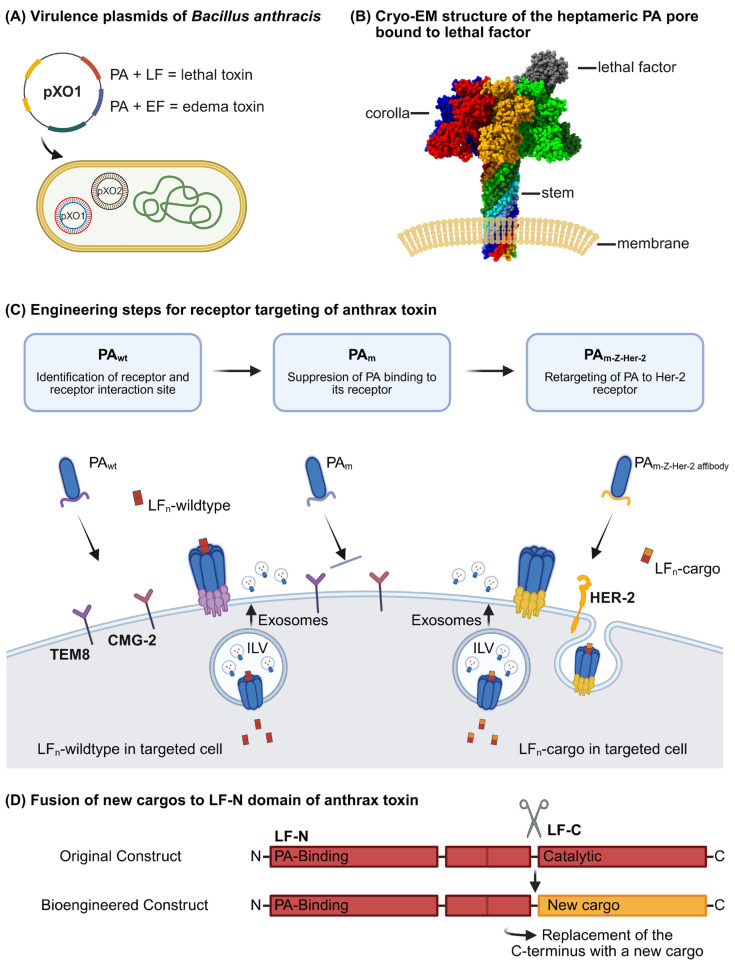
Understanding the mechanisms behind anthrax toxins and their development into a pioneering tool. (**A**) Two plasmids pXO1 and pXO2 are required for full virulence. The plasmid pXO1 encodes three distinct proteins, namely protective antigen (PA), lethal factor (LF), and edema factor (EF). The three proteins assemble into two distinct toxins: lethal toxin, which is composed of protective antigen (PA) and lethal factor (LF), and edema toxin, comprising PA and edema factor (EF) or a mixture. One to three enzymes can be attached to the heptamer. Instead, pXO2 is essential for capsule formation. (**B**) Cryo-EM structure of the heptameric PA pore bound to lethal factor (Accession number PDB database: 6PSN). The PA oligomeric pore enables the translocation of LF or EF into host cells. (**C**) Engineering steps for receptor targeting to specific host cells. First, the two anthrax receptors, namely TEM-8 and CMG-2, were identified and the toxin binding sites subsequently mutated (double-mutated PA (PA_m_)) to inhibit the binding of protective antigen (PA) to its cellular targets. Next, a receptor-targeting motif of interest was fused to PA_m_, as demonstrated by the PA_m_-Z-HER-2 fusion protein, which enables the selective killing of HER-2-expressing cells. Toxin-loaded exosomes may reach additional cells (compare Section 10). (**D**) The N-terminal domain of lethal factor (LF) mediates binding to protective antigen (PA), while the C-terminal domain (LF-C) can be replaced for new cargo proteins. Created in BioRender. Wetterer, G. (2025) https://BioRender.com/r4ozkca.

Anthrax toxins are produced by the spore-forming, Gram-positive bacterium *Bacillus anthracis*. Three separate proteins are encoded on the plasmid pXO1 (Figure 1A): The cell binding and delivery (B) moiety protective antigen (PA), and the two enzymatic (A) components, lethal factor (LF) and edema factor (EF) [1]. The three proteins form two toxins: lethal toxin composed of PA and LF, and edema toxin built from PA and EF. However, since the heptamer can bind one to three enzymes, a mixture of EF/LF can also be attached [2]. Both toxins affect intracellular signaling molecules and thus have to enter the cytosol of mammalian cells. The lethal factor is a zinc-dependent protease which cleaves members of the mitogen-activated protein kinase kinase (MAPKK) family of serine–threonine kinases. As a consequence, important mitogen-activated signaling pathways inducing proliferation are blocked [3]. The edema factor instead is an enzyme with adenylate cyclase activity. Upon stabilization by calmodulin-binding, it leads to the rapid formation of cAMP and activation of protein kinase A [4]. Adenylate cyclase produces cAMP from ATP. Recently, it has been shown that EF-induced ATP depletion is crucial for the EF-mediated toxicity [5]. Protective antigen is needed for cellular binding, endocytosis, and transport of LF or EF into the cytosol. It binds to specific cellular receptors (see below) present on many mammalian cells. On the cell surface, the 83 kDa protein is cleaved by furin and other cellular proteases, allowing oligomerization of the shortened 63 kDa PA (PA63) [6]. LF and EF interact with the oligomer to be internalized by endocytosis. Early biochemical studies suggested that PA63 forms SDS-stable oligomers on cells upon acidification of the culture medium [7,8]. Some years later, the crystal structure of PA83 monomeric protective antigen and of the PA63 oligomer was solved [9]. It indicated that the monomeric PA83 is composed of four domains. The toxin is cleaved within domain one by furin to unlock oligomerization (see Section 4). Loss of the 20 kDa fragment (PA20) of PA83 induces formation of a water-soluble heptamer, which inserts into membranes at the acidic pH present in maturing endosomes [8]. Also, the formation of an octamer in solution and on the cell surface has been described [10,11]. A much clearer picture of pore transition (from soluble prepore to a membrane-inserted pore) could be drawn following the determination of the cryo-EM structure (Figure 1B) [12]. The transition was induced by the acidification of the prepore directly on carbon film-covered grids. The structure showed that residues 275 to 353 from domain two form an extended beta-hairpin following acidification. Seven (or eight) of these hairpins assemble into the membrane-spanning domain of the inserted pore. The inner diameter at the narrowest point of the channel formed is only 6 Å. It is surrounded by ring-shaped phenylalanines, designated as ϕ-clamp. This small diameter suggests that it does not allow for the transition of folded proteins or secondary structural elements. Therefore, bound LF and EF have to unfold during transition and to refold when delivered to the cytosol or into the lumen of endosomal intraluminal vesicles (ILVs), probably with the help of chaperones [13,14].

## 3. Receptors for Cellular Binding

The insertion of proteins into lipid membranes requires close contact amongst them. This is mediated by binding to specific cellular protein receptors. Generally, cellular receptors have been identified for a variety of toxins by biochemical approaches, like mutagenesis or CRISPR Cas-mediated knockout libraries (for review see [15]). Toxins often do not exclusively interact with proteins but also with poly-sugars or lipids to guarantee high-avidity binding. For example, the *Photorhabdus luminescens* toxin complex (PTC, compare chapter 9) has been shown to interact with N-glycans, particularly Lewis X/Y antigens, to facilitate binding to cells [16]. *Pasteurella multocida* toxin (PMT) interacts with the membrane protein low-density lipoprotein-related receptor (LRP1, see below, [17]) and also binds to sphingomyelin [18]. Two cell surface receptors have been identified for the anthrax toxins, namely tumor endothelial marker 8 (TEM-8), designated anthrax receptor 1 (ANTXR1), and capillary morphogenesis gene 2 (CMG-2), also known as anthrax receptor 2 (ANTXR2) [19]. These two proteins share about 40% sequence identity, with more than every second amino acid being identical in the extracellular ligand binding domain. TEM-8 is a transmembrane protein which is upregulated in the vasculature during angiogenesis in response to stimulation by interleukin 1β [20]. Also, CMG-2 belongs to the family of single-pass transmembrane proteins. Both receptors are supposed to mediate cell–matrix interactions and contain extracellular von Willebrand factor A domains which mediate metal-dependent interactions with PA. The affinities between toxin and receptor have been determined in the picomolar range [21,22]. Beyond their role in toxin binding and uptake, the two anthrax receptors mediate tumor cell growth and angiogenesis induced by solid tumors. Therefore, targeting TEM-8 and CMG-2 has been suggested for anti-angiogenic therapy [19]. In addition to the two established anthrax toxin receptors, low-density lipoprotein-related receptor 6 (LRP6) has been suggested as a third component necessary for anthrax toxin uptake. Knockdown of LRP-6, as well as blocking anthrax-LRP-6 interaction by antibodies, inhibits the toxin’s action [23]. LRP-6 was suggested to directly interact with the anthrax receptors and to mediate the endocytosis of the receptor–toxin complex [24]. LRP6 is a key component of the Wnt signaling cascade and regulates activity and localization of GSK3beta and Axin responsible for β-catenin-dependent gene regulation [25]. Interestingly, LRP-related receptor 1 has also been identified to be crucial for the action of bacterial toxins and viruses: various ligands have been identified to interact with LRP1, including viruses like the common cold virus [26] and Rift Valley fever virus [27]. Moreover, bacterial toxins like the *Pasteurella multocida* toxin (PMT) [17], *Clostridium perfringens* TpeL [28], *Pseudomonas* Exotoxin A (ExoA) [29], TcdA produced by *Clostridoides difficile* [30] and the vacuolating toxin (VacA) from *Helicobacter pylori* [31] need LRP1 for binding and endocytosis. The diverse ligands do not contain a common binding motif. LRP1 is suggested to be a general endocytosis or transcytosis receptor [32]. Also, LRP6 might induce endocytosis of TEM-8 and CMG-6-bound anthrax toxin. However, the exact role of LRP6 for binding and uptake of anthrax toxins remains to be determined.

## 4. Structural Analysis of PA and Toxin–Receptor Interaction

The crystal structure of monomeric anthrax PA was solved about 30 years ago [9]. It is composed of four domains mainly formed by beta-sheets. Domain one contains the furin cleavage site located within a disordered loop which is accessible for the protease. Cleavage releases PA20 (amino acids 1–167). This toxin part is not involved in the intoxication process. Instead, it hinders early oligomerization of PA as removal of PA20 exposes a hydrophobic surface in PA63 which enables oligomerization. The crystal structure of the water-soluble heptamer was solved early [9]. The prepore is ring-shaped with a diameter of about 160 Å. Oligomerization is accelerated by receptor clustering with enrichment of receptor–toxin complexes in lipid rafts followed by clathrin-dependent endocytosis [33]. Prepore-to-pore conversion is mediated mainly by multistep structural rearrangements of domain two [12]. Upon acidification, each monomer provides a β hairpin (amino acids 275–353) forming a membrane-spanning 14-stranded β barrel forming the heptameric pore, which is more than 100 Å in length and, therefore, can stretch across a biological membrane. Domains three and four remain peripherally located. Domain four keeps contact with the cellular receptors. Following prepore-to-pore conversion, the channel formed connects the endosomal compartment with the cytosol. It was suggested that the difference in proton concentration and/or electric potential between these two compartments not only is involved in unfolding of the cargo but may also present the driving force for LF/EF translocation [12]. Within the acidified endosomes, proteins are differently charged. The voltage gradient across the organellar membrane may be relevant for protein transport through the channel. No further energy is required. Therefore, PA-mediated transport of the two enzymes may be designated as secondary active transport. In line with this, blocking one of the driving forces with the proton pump inhibitor bafilomycin A slowed down the intoxication of cells [34].

Having learned how PA interacts with mammalian cells and releases their catalytic moiety into the cytosol paved the way to design engineered bacterial protein toxins for the delivery of cargo proteins into cells and to restrict the injection to specific cells by modification of the receptor-binding domain of PA (Figure 1C). The first step in re-targeting the toxin to specific cells was to mutate the intrinsic receptor binding site. This required a tight structural analysis of the toxin–receptor interaction. TEM-8 and CMG2 are type 1 membrane proteins, both containing a metal ion-dependent adhesion site (MIDAS motif) known as a conserved ligand-binding motif in von Willebrand/integrin-A domains. This motif also mediates a divalent-cation-stabilized PA–receptor interaction [35]. Mutation of a specific threonine within this motif inhibits the coordination of the cation and negatively influences ligand binding of integrins. The same was true for mutation of the conserved threonine (T118 in TEM-8) of the anthrax receptors, which diminished PA binding [36]. With regard to the receptor binding site of PA, D683 in PA directly interacts with the bound ion and was identified to be crucial for high-affinity binding to the cellular receptors [22]. In line with this, the mutation of D683 in PA to alanine resulted in about 1000-fold decreased toxicity to mammalian cells [37]. Also, N682 of PA seems important for receptor binding, as substitution with alanine reduced toxicity about 100-fold. Identification of these amino acids was crucial for the development of targeted PA, since natural receptor binding of the respective double mutant PA_m_ (PA (N682A, D683A)) was significantly diminished. This breakthrough finally allowed re-targeting PA to receptors of choice without strong overall toxicity [38,39].

## 5. Re-Targeting PA_m_ to Specific Cells and Tissues

The basis for re-targeting PA was the fusion of heterologous receptor-binding domains to the C-terminal domain four of the double-mutated PA (PA_m_, Figure 1). In first studies, human epidermal growth factor was fused to PA_m_ to allow intoxication of EGF-receptor (EGFR)-positive cells. PA_m_-EGF was expressed, cleaved by furin, capable of forming oligomers, and, together with EF or LF, it intoxicated cells expressing the EGFR. Similar results were reported for a fusion of PA_m_, with the receptor-binding domain of diphtheria toxin able to intoxicate cells expressing the diphtheria toxin receptor [38]. With a view to more clinical applications, the same group developed a PA_m_ fusion construct with a C-terminal human EGF-receptor 2 (HER-2) binding affibody (Z-HER-2). HER-2 is overexpressed in about 25% of human breast carcinomas and associated with aggressive tumor growth. HER-2 is the target of trastuzumab, which is a monoclonal antibody approved for treatment of HER-2-positive carcinomas. Affibodies are stable polypeptides composed of three alpha helices derived from *Staphylococcus aureus* protein A. They can be engineered for high-affinity binding to several proteins without distortion of their structure. The PA_m_-Z-Her-2 fusion protein mediated selective killing of HER-2-expressing cells [39]. Later, PA_m_ was successfully retargeted to epithelial cell adhesion molecule (EpCAM)-expressing cells by C-terminal fusion of a designed ankyrin repeat protein (DARPin) [40]. EpCAM is a cell surface glycoprotein that is highly expressed in epithelial cancers and controls cell–cell interaction in cancer cell migration and in early developmental processes of animals and man [41]. DARPins are artificial protein domains based on the Ankyrin repeat fold consisting of two antiparallel helices, connected three- to five-fold by loops. Like affibodies, DARPins can be selected to bind a given target protein with high-affinity and specificity (for review see [42]). Overall, these results suggest that it is possible to re-target anthrax PA_m_ to different cellular receptors by fusion of receptor-binding domains or peptides. However, loss of PA-Anthrax–receptor interaction in PA_m_ seems to allow oligomerization and pore formation already at physiological pH. This may lead to open pores within the plasma membrane and efflux of ions mediating cellular toxicity at higher concentrations of PA_m_. Therefore, strategies to prevent premature prepore-to-pore conversion on the cell surface are the focus of recent studies [43]. Also, the furin cleavage site was altered to that recognized by matrix metalloproteases (MMPs) or urokinase in order to enhance specificity towards tumor cells by on-site activation. MMPs are highly expressed by cancer cells, which guarantees preferred stimulation of PA oligomerization in close proximity to tumor cells [44].

## 6. The B-Domains as Delivery Mediators for Cargo Transport into Cells

The second goal of anthrax toxin engineering was to exchange the transported cargo. Therefore, the interaction of EF or LF with the PA oligomer on the molecular level had to be studied. Lethal factor comprises four domains with the N-terminal domain being responsible for PA binding [45]. The structural basis for PA–LF interaction and the unfolding of LF for transport through the channel was elucidated by solving the crystal structure of the complex [46]. The crystal structure of the PA octamer bound to the N-terminus of lethal factors (LF-N) revealed that four truncated LF proteins were attached to the PA prepore. In the solved structure, each subunit composed of LF-N bound to a PA dimer had an identical orientation, suggesting that four PA2-LF-N units formed the octamer [46]. In line with this, the heptamer bound a maximum of three LFs in earlier studies [2]. Cleavage of PA by furin seems not only to allow oligomerization but also to support LF binding, unfolding, and transport into the pore. In this process, the N-terminal alpha helix 1 and the first beta sheet of LF-N unfold and dock into a deep pocket of the PA-oligomer, which guides LF-N into the pore; however, the exact mechanism of how unfolding occurs is not known [46]. Since attachment of LF-N to the prepore is sufficient for PA binding and transport into cells, LF-N is usually fused to protein cargo, or to the DNA-binding protein GAL4 for the transport of nucleic acids into cells (Figure 1D) [47,48]. To this end, diverse cargo proteins were fused C-terminally to LF-N and have been successfully transported into mammalian cells by the PA pore [40,49,50,51,52,53]. LF and EF are mainly unrelated proteins. However, five short regions of homology have been observed between the two enzymes within the N-termini responsible for PA binding [54]. Later, it was suggested that the PA-prepore binds nonspecifically to unfolded proteins and that unfolding of LF-N α1, β1 is required for high-affinity binding and transport of proteins through the pore [46].

## 7. Other Binary Toxins for Protein Transport

With anthrax toxin, it has been shown that binary bacterial toxins can be used for the delivery of foreign cargo proteins into mammalian cells, with the B part being sufficient to mediate the transport of proteins through the endosomal membrane, or to deliver the cargo across the ER membrane in the case of retrogradely transported toxins like the *Escherichia coli* heat-labile enterotoxin [55,56,57]. Besides anthrax toxin, the binary C2 toxin produced by *Clostridium botulinum* has also been used for delivery. Like anthrax toxin, C2 is composed of two subunits (C2I and C2II). C2I is an ADP-ribosyl-transferase which modifies actin [58]. C2II is similar to anthrax PA. It is proteolytically cleaved, a prerequisite for oligomerization, C2I-binding, and insertion into biological membranes [59,60]. As described above for anthrax toxin, acidification is required for translocation of the ADP-ribosyl-transferase and for the delivery of cargo molecules fused to the catalytically inactive N-terminal part of C2I (C2I-N) [56,61,62]. Recently, it has been shown that the delivery of cargos by the C2II oligomer is more efficient for proteins containing a positively charged N-terminus [63]. In addition to anthrax toxin and C2 toxin, the binary toxin CDT from *Clostridoides difficile* has also been utilized for protein cargo transport [64]. Again, the N-terminal portion of the natural enzymatic component is required for binding to the translocation part, and a cargo protein is fused to it. There are further known binary toxins with a similar uptake mechanism which may be developed for this purpose: iota toxin produced by *Clostridium perfringens*, *Clostridium spiroforme* toxin [65,66,67,68,69], and heat-labile enterotoxin from *Escherichia coli* [70].

## 8. Single-Chain Toxins for Delivery of Cargo Proteins

The uptake mechanism of single-chain AB toxins into mammalian cells is different from binary toxins. However, they likewise possess the intrinsic ability to enter the cytosol of mammalian cells and can be engineered as elegant nanomachines for the delivery of cargo proteins [71]. They possess a modular structure with at least three domains: a catalytic domain, a receptor-binding domain and a so-called translocation domain present in a single protein. The translocation domain is necessary for the delivery of the catalytic domain into the cytosol of cells. It is generally a centrally located part, whereas the catalytic domain and receptor-binding part can be either found N-terminally or located at the C-terminus, respectively. The actual view of how single-chain toxins enter the cytosol of mammalian cells is as follows: the toxins bind to one or several receptors on the cell surface and are taken up by receptor-mediated endocytosis. “Short-trip” toxins (like Diphtheria toxin) are directly released from endosomes into the cytosol after acidification. Partial unfolding of the proteins may uncover hydrophobic amino acid patches required for insertion into the endosomal membrane. It is still not fully understood how exactly these toxins mediate the delivery of their catalytic part into the cytosol. Therefore, the precise requirements for the proteins to be transported are difficult to elucidate. “Long-trip” toxins (like Cholera toxin) take the route back through the Golgi apparatus and endoplasmic reticulum. From here the toxins are released into the cytosol by the host endoplasmic-reticulum-associated protein degradation machinery (ERAD) which physiologically transports misfolded proteins to the cytosol for the re-use of amino acids. To avoid the intrinsic toxicity of the carrier-toxins, the catalytic part has been completely removed or catalytic amino acids have been exchanged to generate a catalytically inactive A-domain. Single-chain toxins which have already been engineered for cargo transport include Diphtheria toxin [72,73,74,75], different botulinum neurotoxins delivering cargo proteins to neurons [75,76,77,78,79], and Pseudomonas exotoxin A [80]. In contrast to the uptake of binary toxins, the exact mechanism of translocation of single-chain toxins through biological membranes remains to be fully elucidated.

## 9. Injection Machineries

**Figure 2:**
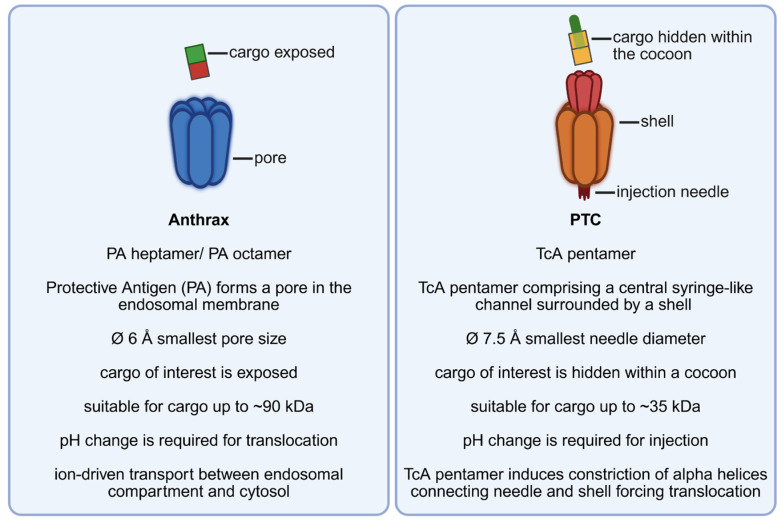
A mechanistic comparison of anthrax toxin and the Photorhabdus toxin complex (PTC): similarities and differences.

Syringe-like toxin complexes (TCs) are produced by insect pathogens like *Photorhabdus* and *Xenorhabdus* species to inject toxins directly into insect cells [81,82,83,84]. Some of the TCs bind to insect as well as to mammalian cells. The injection machines solely formed by proteins are composed of three subunits: TcA, TcB and TcC [85]. These come together in a 5:1:1 (A:B:C) stoichiometry and shape a high-molecular-mass (~1.7 MDa), tripartite ABC-type holotoxin [86]. Oligomerization of TcA to a homo-pentamer results in the formation of a large (~1.4 MDa) structure that contains a central syringe-like channel, which is surrounded by a shell [87]. This toxin part is required for cell binding and injection of Tc toxins. In contrast to anthrax toxin, these enzymes are not freely exposed to the surrounding fluids but congregate into a cocoon-like complex composed of TcB and TcC. This cocoon-like structure, with the enzyme packed inside, interacts with the top of the injection channel to complete the holotoxin [88,89]. The enzymatic part of PTCs is located at the C-terminus of TcC [89,90]. The toxic enzyme is autoproteolytically separated from the TcC N-terminus to be injected through the needle [86]. In engineered *Photorhabdus* toxin complexes (PTCs), this C-terminal part of TcC has been exchanged for diverse cargo proteins, like *Clostridium botulinum* C3 toxin or a luciferase, for quantitative analysis of the number of injected proteins. All these artificial proteins can be delivered into living mammalian cells [91,92]. Due to functional delivery, the cargo seems packed into the cocoon already during recombinant expression of the fusion protein. Thus, the size of the cargo seems limited by the space within the cocoon. Like shown for anthrax and single-chain bacterial toxins, pH changes are required for the translocation of the enzyme/cargo. For PTCs the basic nature of the insect gut or acidification of maturing endosomes in mammalian cells likewise induces conformational changes within the TcA pentamer, inducing the constriction of alpha helices which connect the “needle” with the “shell” [93]. The loaded enzyme (or cargo protein) is thereby injected into the cytosol [94,95,96]. A recent publication analyzing prepore-to-pore transition suggests a tip diameter of about 7.5 Å, which is close to the smallest diameter of the anthrax pore (6 Å) and therefore likewise needs unfolding of the cargo to be transported [97] (Figure 2).

## 10. A Critical View on the Application of Targeted Protein Toxins

With anthrax toxin as a pioneer, several bacterial toxin-based systems have been utilized to deliver proteins of interest into the cytosol of mammalian cells. In the nervous system of the mouse, a naturally high expression of ANTXR2 has been found on pain-sensing Nav1.8+ DRG neurons (nociceptors) with only marginal expression of anthrax receptor 2 on CNS neurons. Recently, it has been shown that intrathecal administration of un-modified edema toxin induced analgesia in mice without harmful effects for CNS neurons. In this study, engineered anthrax toxins have also been used to transport cargo proteins like botulinum toxin into nociceptor cells in vivo [98]. This shows that anthrax toxin can be used in living organisms. Selective targeting of cells combined with the ability to deliver cargo molecules into the cytosol is common in all toxin-based delivery systems developed. Toxins use various molecular mechanisms of protein translocation, ranging from single-chain toxins via pore-forming toxins to injection machineries. In each case, modification or replacement of the receptor-binding domains and mutation of catalytic amino acids or removal of the catalytic domains enabled scientists to restrict overall toxicity. However, cell-to-cell spreading of anthrax toxin may occur by exosomes so that cells without the anthrax receptor can also be reached. This process requires ALIX and other ESCRT proteins [14]. The toxin’s mechanism of uptake seems more complex than originally assumed. Recent analysis has shown that following endocytosis EF/LF is additionally transported through the membrane of intraluminal vesicles (ILVs) within the endosomes. This mechanism allows long-term storage of the native toxin in ILVs (for days), since the endo-ILV milieu resembles the neutral one of cytosol. Moreover, EF/LF-loaded ILVs have been shown to be delivered to other cells and tissues as exosomes (compare Figure 1). Exosomes can be taken up by naïve cells independent of the expression of the anthrax receptors, which may be problematic for targeted toxins [14]. The clinical use of these engineered toxins is still challenging. As foreign proteins formation of neutralizing antibodies against them is likely and, therefore, repeated application as potential biological drugs may be restricted. Therefore, reducing immunogenicity is required as a next step of development. As learned from the clinical use of antibodies, it is possible to replace antigenic surface epitopes with inert ones. Proteases present in body-fluids may destroy the protein toxins before they interact with the target cell. This is expected, especially if the engineered toxins are used in an environment usually not reached by the original toxins. In contrast to targeted toxins, clinically used antibodies are protected from degradation by binding to the neonatal Fc-receptor (FcRn) and thus have a long-lasting half-life. Besides attack by the host, the biggest limitation of a future clinical application of engineered bacterial toxins is probably their restricted distribution in tissues. However, many anthrax-toxin-based clinical studies and trials using other engineered bacterial toxins have been performed. Their findings are summarized in an excellent recent review by Misra et al. [99]. Many milestones in understanding the action of bacterial toxins for the purpose of re-targeting them have been reached. However, further work is required to establish a reasonable set of engineered bacterial toxins for safe and efficient clinical use.

## Data Availability

No new data were created or analyzed in this study.
